# Speech Outcomes after Tonsillectomy in Patients with Known Velopharyngeal Insufficiency

**DOI:** 10.1155/2012/912767

**Published:** 2011-11-22

**Authors:** L. M. Paulson, C. J. MacArthur, K. B. Beaulieu, J. H. Brockman, H. A. Milczuk

**Affiliations:** Division of Pediatric Otolaryngology, Department of Otolaryngology/Head and Neck Surgery, Division of Speech and Language Pathology, Doernbecher Children's Hospital, OHSU Portland, OR 97239, USA

## Abstract

*Introduction*. Controversy exists over whether tonsillectomy will affect speech in patients with known velopharyngeal insufficiency (VPI), particularly in those with cleft palate. *Methods*. All patients seen at the OHSU Doernbecher Children's Hospital VPI clinic between 1997 and 2010 with VPI who underwent tonsillectomy were reviewed. Speech parameters were assessed before and after tonsillectomy. Wilcoxon rank-sum testing was used to evaluate for significance. *Results*. A total of 46 patients with VPI underwent tonsillectomy during this period. Twenty-three had pre- and postoperative speech evaluation sufficient for analysis. The majority (87%) had a history of cleft palate. Indications for tonsillectomy included obstructive sleep apnea in 11 (48%) and staged tonsillectomy prior to pharyngoplasty in 10 (43%). There was no significant difference between pre- and postoperative speech intelligibility or velopharyngeal competency in this population. *Conclusion*. In this study, tonsillectomy in patients with VPI did not significantly alter speech intelligibility or velopharyngeal competence.

## 1. Introduction

Patients with velopharyngeal insufficiency (VPI) present a unique challenge to the pediatric otolaryngologist. Otherwise straightforward problems such as sleep apnea and chronic tonsillitis become far more complicated in the cleft population due to the risk of VPI, and yet, sleep disordered breathing is increasingly being diagnosed in this group. Several recent studies demonstrated that cleft patients exhibit a significantly higher incidence of sleepdisordered breathing, with syndromic patients carrying an increased risk for obstructive sleep apnea (OSA) [1, 2]. However, OSA tends to be underdiagnosed and undertreated in this population, which may be due to a reluctance to operate in the oropharynx due to risk of velopharyngeal insufficiency.

Adenoidectomy has long been known to carry a risk of worsening velopharyngeal insufficiency in patients with known VPI and may unmask previously undiagnosed VPI, particularly in patients with submucous cleft palate [3–6]. However, controversy exists over whether tonsillectomy alone will affect speech in patients with known velopharyngeal insufficiency, particularly in those with cleft palate.

In this study we hypothesize that, in carefully chosen patients, tonsillectomy can be safely performed on patients with existing VPI without significant adverse effects to their speech.

## 2. Methods

All patients seen at the Doernbecher Children's Hospital Multidisciplinary Velopharyngeal Insufficiency Clinic between 1997 and 2010 were prospectively entered into a database. The database was screened for all patients undergoing tonsillectomy during this time. Inclusion criteria included a previous diagnosis of velopharyngeal insufficiency, a history of tonsillectomy performed at this institution, and adequate pre- and postoperative speech assessment. Exclusion criteria included concordant adenoidectomy, pharyngoplasty or pharyngeal flap, or inadequate speech evaluations for analysis.

Speech assessments were obtained from routine speech analysis by pediatric speech pathologists, on the scale developed at this institution prior to the acceptance of the universal speech parameters. The analysis was translated to a nonparametric scale as indicated below in Tables 3 and 4. Those patients who underwent pre- and postoperative nasal endoscopy were graded on the Golding-Kushner scale [7] on palatal motion and lateral pharyngeal wall motion as judged by a single faculty pediatric otolaryngologist (H. A. Milczuk). 

Attention was specifically turned to the assessment of two primary parameters for analysis: speech intelligibility and velopharyngeal sufficiency. Pre- and post operative evaluations were compared. For individual patients, a significant change in function was defined to be a change in 2 points on the scale. For the overall group, a two-tailed Wilcoxon rank-sum test was used to evaluate for significance.

## 3. Results

A total of 46 patients with known VPI underwent tonsillectomy over this time period. Of these, 23 had both pre- and postoperative speech evaluations that were sufficient for our analysis. See Table 1 for patient characteristics. The majority of these patients (87%) had a known history of cleft palate. Primary Indications for tonsillectomy included obstructive sleep apnea in 11 (48%), staged tonsillectomy prior to pharyngoplasty in 10 (43%), recurrent strep tonsillitis and VPI in 1 (4.3%), and obstructive dysphagia in 1 (4.3%). Average tonsillectomy size (as graded on a 0–4 + scale) was 3.6 with a median value of 4.0, indicating a strong preponderance of tonsillar hypertrophy in this group.

Overall there was no statistically significant difference between pre- and postoperative speech intelligibility (with trend towards improvement, *P* = 0.13) or velopharyngeal competency (no trend *P* = 0.83) in this population using the Wilcoxan rank-sum. With respect to speech intelligibility, one patient demonstrated significant worsening, and 2 patients demonstrated significant improvement as defined by a change in 2 points on the scale. With respect to velopharyngeal insufficiency, only one person had significant worsening of their insufficiency. No significant trends were noted when comparing patient outcomes with respect to cleft type, age of patient, gender, indication for surgery, or tonsil size.

Ten of the patients underwent both pre- and posttonsillectomy nasal endoscopy, and their data is presented in Table 2. Palatal closure remained the same in 5 (50%), improved in 3 (30%), and worsened in 2 (20%). Lateral wall movement remained the same in 3 (30%), improved in 5 (50%), and worsened in 2 (20%).

## 4. Discussion

The role of tonsils on velum position and function is poorly characterized. The velum position during speech depends on the complex balance of vector forces created by palatal elevators, depressors, and constrictors [8]. Elevation is primarily achieved by the levator veli palatini, and transverse closure is mediated primarily by the superior constrictor. The palatopharyngeal and palatoglossal muscles, between which the palatine tonsils reside, serve to depress the palate. The position of these arches can vary based on the size and shape of the tonsils, and, theoretically, the vector forces exerted on the palate may be affected. While there are many theories and practices, the effect of tonsillectomy in patients with VPI is largely unknown, yet there remains widespread hesitancy to perform tonsillectomy in these patients.

Few studies have characterized the influence of tonsils on speech. A 1994 study by Finkelstein et al. examined tonsil size and position in relation to speech function. They concluded that in most cases markedly enlarged tonsils do not appear to affect velopharyngeal closure as they are typically positioned below the level of velum closure [9]. However, several case reports have described VPI associated with patients with superior pole hypertrophy in which the pole extends above the level of the velum. Endoscopic exam in these cases revealed prominent superior poles which appear to contribute to VPI by extending into the lateral ports, thus, displacing the palatopharyngeus muscle anteriorly [9–11]. It is argued that tonsillectomy may play a role in improving VPI in such cases.

On the opposite side of the spectrum, certain cases of VPI may be expected to worsen with tonsillectomy. In some, the tonsils are thought to act as lateral obturators, particularly in patients who have had pharyngeal flaps that are narrow or low placed. In these cases tonsillectomy would not be recommended or may necessitate simultaneous or staged flap augmentation or revision [12]. These cases should be identified with careful endoscopy. Thus, the role of preoperative endoscopy is emphasized.

In our study, patients with VPI who underwent tonsillectomy had very little overall change in speech parameters. Our findings are consistent with several studies. D'Antonio et al. in 1996 demonstrated improved or unchanged speech parameters in 15 patients at risk for VPI after tonsillectomy [13]. 

Similar results have been demonstrated in other groups. In a recent Taiwanese study by Hu et al. comparing management of VPI in the presence of tonsillar hypertrophy, a subset of patients who underwent an isolated tonsillectomy either alone or for staged pharyngoplasty had similar speech outcomes to our study. In their study, 19 of the patients underwent tonsillectomy without a simultaneous velopharyngeal procedure. Of these, 14 patients had no change in function, three patients improved, and two patients worsened after tonsillectomy [14]. 

Potential biases of this study include observer bias and selection bias. The evaluators were not blinded to the pre- versus postoperative status in these cases. The selection of patients for surgery was not randomized and was based on clinical judgment. An argument against this bias in this study, however, is the inclusion of the subset who had severe VPI and underwent tonsillectomy as a first stage prior to definitive pharyngoplasty. In these patients it was felt that a procedure to improve speech, which invariably narrows the pharynx, would likely result in sleep-disordered breathing postoperatively. These patients arguably may have been the most “at risk” for worsening speech parameters given the preoperative decision that the patient would need VPI surgery. However, this group did not have significantly different outcomes.

It should be noted that the average tonsil size in this study was large (3−4 + in the majority of patients). Thus, the results must be interpreted with respect to this. Studies of tonsil size and speech characteristics are sparse. In one study of healthy male adults, Mora et al. demonstrated that tonsil size was directly related to the degree of audible speech changes after tonsillectomy, notably, the degree in change of improvement of hyponasality [15]. However, data on velopharyngeal competency with respect to tonsil size is sparse.

Furthermore, we must address the fact that only half of the patients in our VPI database who underwent tonsillectomy had adequate pre- and postoperative speech evaluation with standardized perceptual speech analysis. We excluded those patients with speech assessments that did not quantify the two parameters of interest, namely, speech intelligibility and velopharyngeal competency. Several patients also were lost to followup or had a delay in postoperative evaluation, such that a direct comparison of pre- and postoperative speech parameters would not be useful. Ideally, a study of this design should have standardized time intervals for speech evaluation.

## 5. Conclusion

In this study, tonsillectomy without adenoidectomy in patients with VPI and tonsillar hypertrophy did not significantly alter speech intelligibility or velopharyngeal competence. This must be interpreted with respect for adequate clinical judgment. More research is needed to further elucidate the impact of tonsillectomy on patients with or at risk for velopharyngeal insufficiency, particularly given the high prevalence of OSA in this population.

## Figures and Tables

**Table 1:** Patient characteristics.

Patient	Sex	Age at tonsil surgery	Tonsillectomy indication	Tonsil size	CP type	Initial cleft repair	Speech intelligibility			VPI severity		
							Preop.	Postop.	Diff	Preop.	Postop.	Diff
1	F	4	OSA	3.5	UC	2 flap	4	2	−2	3	2	−1
2	M	4	OSA	4.0	BC	push back	1	1	0	0	2	2
3	M	5	OSA	4.0	UC	2 flap	2	1	−1	1	1	0
4	F	12	VPI	3.0	SM	Unk.	2	2	0	2	2	0
5	M	6	VPI	3.5	None	None	2	2	0	3	2	−1
6	M	8	VPI	4.0	UC	2 flap	3	2	−1	2	2	0
7	M	5	VPI	3.0	UC	2 flap	3	2	−1	1	2	1
8	M	8	OSA	3.5	UC	Unk.	0	0	0	1	1	0
9	M	6	VPI	3.0	UC	2 flap	3	4	1	4	4	0
10	M	7	VPI	2.5	SM	Furlow	3	1	−2	3	2	−1
11	F	4	OSA + VPI	4.0	None	None	1	4	3	2	3	1
12	F	5	VPI	4.0	UC	2 flap	4	4	0	4	4	0
13	M	7	OSA	4.0	UC	2 flap	3	2	−1	1	1	0
14	F	7	OSA	3.0	BC	Push back	1	1	0	2	1	−1
15	M	7	Strep+VPI	4.0	I	2 flap	2	1	−1	1	2	1
16	F	7	VPI	2.5	None	none	3	3	0	3	3	0
17	M	7	OSA	4.0	UC	2 flap	2	2	0	2	2	0
18	F	12	OSA+VPI	4.0	I	Furlow	3	2	−1	2	1	−1
19	M	4	OSA	4.0	I	Unk.	3	2	−1	0	1	1
20	F	8	Dysphagia	4.0	I	Unk.	0	0	0	0	1	1
21	F	7	OSA	4.0	UC	Unk.	1	1	0	1	1	0
22	F	8	VPI	3.5	SM	None	4	4	0	3	3	0
23	M	4	OSA	3.5	UC	2 flap	2	2	0	2	1	−1

Patient characteristics. Cleft type: UC: unilateral complete, BC: bilateral complete, SM: submucous, I: incomplete.

**Table 2:** Nasal endoscopy scores.

Pt.	Indication for tons.	CP type	Initial cleft repair	Protons. endoscopy	Posttons. endoscopy	Speech intelligibility			VPI Severity		
						Preop.	postop.	Diff.	Preop.	Postop.	Diff.
4	VPI	SM	Unk.	Palate 9 lat 4	Palate 9 lat 4	2	2	0	2	2	0
5	VPI	None	None	Palate 7 lat 2	Palate 9 lat 4	2	2	0	3	2	−1
6	VPI	UC	2 flap	Palate 0 lat 0	Palate 0 lat 1	3	2	−1	2	2	0
7	VPI	UC	2 flap	Palate 9 lat 2	Palate 9 lat 1	3	2	−1	1	2	1
9	VPI	UC	2 flap	Palate 1 lat 1	Palate 2 lat 1	3	4	1	4	4	0
12	VPI	UC	2 flap	Palate 5 lat 1	Palate 5 lat 3	4	4	0	4	4	0
15	Recurrent strep + VPI	I	2 flap	Palate 8 lat 4	Palate 9 lat 4	2	1	−1	1	2	1
16	VPI	None	None	Palate 9 lat 4	Palate 7/8, lat 1	3	3	0	3	3	0
17	OSA	UC	2 flap	Palate 8 lat 0	Palate 8 lat 1	2	2	0	2	2	0
22	VPI	SM	furlow	Palate 4 lat 0	Palate 3.5 lat 1	4	4	0	3	3	0

Patient characteristics. Tons: tonsillectomy. Cleft type: UC: unilateral complete, BC: bilateral complete, SM: submucous, I: incomplete. Unk.: unknown. Endoscopy scores: palate: palatal movement, lat: lateral wall movement. Scores according to the Golding-Kushner Scale, adjusted to 0–10 scale.

**Table 3:** Ranking of speech intelligibility.

Speech intelligibility		
0	Normal	100% intelligible
1	Minimal	95–99% intelligible
2	Mild	80–94% intelligible
3	Mod	50–79% intelligible
4	Severe	<50% intelligible

**Table 4:** Ranking of velopharyngeal insufficiency.

Velopharyngeal insufficiency	
0	Normal
1	Minimal
2	Mild
3	Mod
4	Severe
